# The perceptions of older people living with hiv/aids towards physical activity and exercise

**DOI:** 10.1186/s12981-022-00500-0

**Published:** 2022-12-27

**Authors:** Levin Chetty, Saul Cobbing, Verusia Chetty

**Affiliations:** Discipline of Physiotherapy, School of Health Sciences, University of KwaZulu-Natal, Westville Campus, Private Bag X54001, Durban, 4000 Westville South Africa

**Keywords:** HIV/AIDS, Physical activity, Rehabilitation, Ageing

## Abstract

**Background:**

Older people living with HIV (OPLWH) require significant levels of support, including healthcare and rehabilitation interventions. People living with HIV are living longer, but still experience health-related impairments that affect functional activity, participation in day-to-day interactions, livelihoods and overall quality of life. Physical activity and exercise should be included as part of the comprehensive medical management for OPLWH but the investigation of prior studies reveal a gap in understanding and prescription. Our study aimed to explore the perceptions of OPLWH about physical activity and exercise.

**Methods:**

The study adopted a phenomenological, qualitative design, using in-depth interviews, to understand OPLWH perceptions of physical activity and exercise, and their need for, and access to, physical activity and exercise programmes in a community in South Africa. Nine [9] males and seven [7] females participated in the study.

**Results:**

Sixteen individuals voluntarily participated in face-to-face, semi-structured interviews which took place at the healthcare facility where they received regular treatment. All participants were 50 years and older. Personal gratification and the ability to perform activities of daily living as well as participate in community activities were believed to be strong motivators for exercise participation, while barriers to exercise were attributed to physical health issues; lack of proper instruction as well as stigma associated with HIV status within their communities. Participants also favored a combination of aerobic, flexibility and strength activities, as well as proper supervision and instruction within a group exercise setting.

**Conclusion:**

The qualitative nature of our study provided an in-depth understanding of the perceptions of OPLWH towards physical activity and exercise. Our study highlighted the factors that hinder adherence to physical activity and exercise in this population. Many indicated that they would love to engage in structured physical activity programmes, but did not know where, when or how to begin. Creating a suitable environment with proper supervision and instruction by suitably qualified health professionals are essential when developing a community-based exercise programme for OPLWH.

## Introduction

Antiretroviral therapy (ART) has increased the longevity of people living with HIV [1]. People living with HIV are living longer, but at the same time still experience health-related impairments that affect functional activity, participation in day-to-day interactions, livelihoods and overall quality of life [2]. Impairments can include both cognitive declines and deterioration in physical health. These health-related limitations and impediments to daily function can be associated with infection; with the side effects of the treatment regimen and, in the older population, the natural effects of ageing on the immune system [3]. These HIV-related impairments, leading to functional challenges and participation restrictions, often lead to disabilities experienced by people ageing with HIV [4].

Despite the high prevalence of disability experienced by people who are ageing with HIV, many HIV-specific care centres do not include adequate rehabilitation and, as a result, many older people living with HIV (OPLWH) cannot access these services or programmes [5]. Disability in older people living with HIV can be described as the fluctuating physical, mental and social health-related challenges, including symptoms and any prevalent impairments; challenges with the activities of daily living (ADLs); and challenges relating to inclusion within their families and the communities they live in [6]. Rehabilitation in the form of physical activity and exercise has a critical role to play in addressing disability and promoting quality of life among OPLWH [7]. Rehabilitation, in this context, is mainly aimed at addressing impairments, activity limitations and participation restrictions often endured by this population, in order to improve their quality of life.

OPLWH require significant levels of support, including healthcare and rehabilitation interventions. While some may continue to live without the need for additional health and financial services or social care, there are many people growing older with HIV who, now and in the future, may require significant levels of support and rehabilitation interventions [1]. With reductions in public health and social care budgets, the future is very uncertain throughout the world; but more specifically in low-to-middle-income countries, which lack adequate support and resources for this population [8].

As people age with HIV, the prescription of physical activity and exercise should be included as part of their comprehensive medical management [9]. Regular physical activity and exercise can help to lower the risk of falls, improve physical and mental health and well-being, strengthen social ties, and improve cognitive function [10, 11]. From an HIV perspective, it can improve cardiovascular fitness, strength, body composition and overall quality of life [12–14]. However, growing older with HIV, coupled with a lack of physical activity and exercise, can significantly accelerate declines in physical, mental and functional status [15].

Physical activity and exercise can be used to improve overall function and quality of life in older populations living with HIV [16]. Physical activity and exercise participation also appear to be a safe and effective way to prevent the negative health outcomes associated with comorbidities in older people living with HIV [17]. In this specific population, alone, physical activity and exercise can be diverse and can focus on health-related challenges that are associated with musculoskeletal, cardiovascular, neurological or multi-system health conditions [18]. Physical activity and exercise can further maximise function and improve overall quality of life in older people living with HIV. Physical activity is a necessity and its importance cannot be overlooked; but despite its importance, high rates of sedentary behaviour are common in older people living with HIV.

In this study we explore the perceptions of older people living with HIV about physical activity and exercise. Exercise was defined as participation in any structured form of physical activity either individually or within a group [19]. Within the context of HIV, understanding the barriers to, and perspectives of physical activity and exercise among older people living with HIV is essential in order to contribute to feasible, tailored programmes for their participation in physical activity and exercise [20]. Furthermore, understanding the perceptions of older people living with HIV about physical activity and exercise is essential in prescribing physical activity and exercise for this population and influences the uptake of these programmes. Exploring their attitudes, and understanding their thoughts about physical activity and exercise, will also assist in distinguishing between the viewpoints of participants with, and without, established exercise habits. The identification of the influences on exercising or routine physical activity, such as physical health and self-efficacy, and accessibility to safe environments for exercise, is also an imperative [21]. It is important to identify the facilitators of, barriers to and suitable environments for physical activity and exercise, specific to older adults living with HIV taking context into consideration.

## Methods

### Design

The study adopted a phenomenological, qualitative design, using in-depth interviews, as a way to understand the participants’ perceptions of physical activity and exercise, and their need for, and access to, physical activity and exercise programmes. This process of gaining insight into the experiences and perceptions of older people living with HIV allowed the researchers to identify barriers to, and enablers of, physical activity and exercise in OPLWH. This phenomenological approach, using interviews, involved an informal interactive process that aimed to elicit a personal, comprehensive description of the lived experiences of this phenomenon in OPLWH [22].

### Participant selection

Participants for the study were purposively sampled using a convenience sampling technique. They included male and female participants aged fifty [50] years and older who receive healthcare at the study setting. Following the rigorous process of telephonically inviting participants from this semi-rural area in KwaZulu-Natal, South Africa, to the interview, nine [9] male and seven [7] female participants voluntarily consented to participate in our study. All the participants met the study’s inclusion criteria of being fifty years and older, living with HIV, and on ARTs.

### Study setting

The interviews were conducted in a private seminar room in the Physiotherapy Department within the healthcare facility. The study setting, a 200-bed semi-rural, level-one district public healthcare facility, is situated on the outskirts of the eThekwini Municipality in the province of KwaZulu-Natal. It serves a population of approximately one million people and acts as a referral hospital for sixteen provincial and municipal primary health clinics, as well as two community health centres [CHCs]. The hospital provides a service for approximately 4 500 people living with HIV, whilst estimates indicate that more than 250 000 people [33%] living in the catchment area are HIV-positive.

### Data collection

Informed consent for participation in our study was obtained and permission to use audio recordings was granted by each OPLWH. Each participant was informed that participation was voluntary, and of their right to withdraw at any time. No financial renumeration was provided to participate in the study as we conducted the interviews following their routine clinic visit. Participants were given the option to converse in isiZulu or English. isiZulu was the preferred choice, as it is the common first language of the local community, with about 12000 000 native speakers, primarily in the province of KwaZulu-Natal, in South Africa. The Zulu recordings were subsequently translated into English by a qualified language interpreter. The recordings were back-translated to ensure rigour. The interview was guided by the following questions; what is your understanding of exercise and/or physical activity; what is your understanding of exercise and/or physical activity and HIV; do you participate in exercise or physical activity; how many times a week do you exercise, how long is each exercise session, do you exercise at home or go to a gym/hospital/facility; what type of exercise do you partake in each session; do you enjoy exercising; how has exercise benefited you in your day to day activities; what factors prevents you from exercising. An interviewer and an expert moderator in qualitative research were present during the interviews with each participant. Each interview lasted approximately sixty [60] min.

### Data analysis

The responses from the OPLWH were transcribed verbatim immediately after the interviews. Transcripts were read and re-read by two of the researchers independently to gain an understanding of the composite narrative. An inductive approach was used to analyse the data, thereby allowing the researchers to analyse the data using a bottom-up approach, ensuring that codes and themes were derived from the content of the data itself. Braun and Clarke’s open coding process was followed to identify common concepts in the participants’ responses. As outlined by Braun and Clarke, the six-step approach to thematic analysis included: (1) familiarising oneself with the data; (2) the generation of initial codes; (3) searching for themes; (4) reviewing the potential themes; (5) defining and naming the themes; and (6) producing the report [23]. The researchers debated the codes and categories until final themes were agreed upon. Member checking was conducted to verify the accuracy of the data transcription. In order to gain an articulate, comprehensive view of the phenomenon, the process of method triangulation was used throughout each phase to ensure the credibility and trustworthiness of the data [24].

## Results

Participants took part in a face-to-face, semi-structured interview [each approximately 60 min in length] between June and November 2021. There were sixteen [16] individuals who voluntarily participated in the interview process. All participants were 50 years and older, and living with HIV. Nine [9] were males and seven [7] were female. Participants were predominately black South Africans, with the exception of one [1] female participant who was of mixed race. In South Africa, the term ‘coloured’ is used to define individuals of mixed race. The average age of the participants was 64 years for the men and 60 years for the women. The number of years of living with HIV ranged from eight years for the male participants, and 12 years for the female participants. Eleven [11] of our participants were not retired and were still actively contributing to their community. While most participants reported engaging in some form of physical activity and exercise every now and then in the past year, only six [6] participants reported being actively involved in a structured exercise regime for approximately twenty [20] min or more each day or on alternate days during the week.

The data revealed three [3] overarching themes that emerged from our interview process, namely: motivation for physical activity and exercise; barriers to physical activity and exercise; and proposed structure of physical activity and exercise. The themes, subthemes and illustrative quotes of participants are presented in Table 1.

**Table 1:** Themes, subthemes and illustrative quotes of participants

Theme 1: Motivation for physical activity and exercise	
Recommendation by health professional	“Well, my doctor mentioned that I must be physically active but I don’t usually do that but I never sit at home. I’m always working, I farm. Exercise is not something that I choose to do.” “I realise that walking and things, it’s quite important if I do go to the centre in Pinetown. I don’t take the lifts. I use the stairs and I find that’s important because last year I had to undergo a major operation and the doctor wanted to know, do you ever go up a flight of stairs instead of the lift? It’s really important.” “My doctor only advised me to remain physically active and not to allow myself to sit most of the time so that I don’t experience most of the negative effects of this sickness.”
Personal gratification	“Well I never thought about it, but it’s something I don’t think I can shy away from, because it will add another value to my life. I believe that psychologically it works you up because if you are 24/7 at home you keep on thinking of the small minor stupid things. And then whatever you are doing in order to boost yourself because of the worries that you have, then it takes a big toll. By the time you take tablets if ever you are taking tablets, they are not doing much because you are still stuck in that mystery that you are thinking about, you know. But maybe exercises help you to become fresher. I would say that it is a good thing to do because, as I’ve said, you won’t keep on thinking about stupid things most of the time because once you take tablets and then sometimes you are not going anywhere you sit down, then all these things are coming back but maybe if you are doing exercise, it goes away.” “Without a doubt (referring to exercise) that’s the one reason I am still living today.” “No one advised me on exercising. I took it upon myself to be physically active because I saw the need to. When I take my walks, I come back home feeling stronger and lighter and I’m able to spend more time in the garden doing some farming till it becomes dark outside.”
Activities of daily living as a driver	“Yes, I love farming. I have chickens at home. I wake up in the morning and clean the house and take care of the chickens. I’m very active. Remove the weeds and dirt in the garden. I water the garden as well and clean up after the chickens as well.” “Weekly I’ve got a job at home. I’m doing my garden with my wife. Yesterday we were cleaning up the potatoes and today I’m going for my beans and tomorrow morning I’m going up to my daughter’s house in Pretoria to plant beans for my daughter because only me and my wife work in the garden. My daughters and sons know nothing about the garden. So, my job is to do the garden.” “Anytime in the day I work in my garden. I can go home now and go work in my garden. Later on I watch the news at about 13:00. After 13:00 I come back in feed my fowls, clean the yard. Every day, I must feed my fowls. I look out for my goats. I walk around and see down in the garden and pull the wires and the poles.”
Community participation in a religious context	“Yes, it does happen that I walk long distances, especially on Sundays where I attend church. It’s very far from home.” “At my church we do run around. I do a lot of running at church, worshipping at a Zion church. I definitely run. Everyone participates in church” “A lot of work, yes. I even play the drums there or clap my hands as a form of instrument.”
Family responsibility and influence	“I would also keep very active when my grandchild was alive. I would bath him and exercise with him.” “Yes exactly, my granddaughter, she does soccer so she trains most of the time, so when she’s at home to do what she does, she finds me lying (down) and she will… you know… she sorts of helps me in lot of things. I love to exercise with her.” “I’m active and I wake up early. I live with my grandchildren and I always take them to school in the morning. One of my grandchildren, who is eight years old, I give her a bath and prepare her myself for school. I don’t mind working till the sun sets—I have no problem at all.”

Theme 2: Barriers to physical activity and exercise	
HIV-related stigma	“Okay, and then in hindsight, say that you do have the facilities for exercise and people outside, they will be able identify that the people who are exercising here are the people who are HIV-positive. Well actually, I for one, even though this is something at times I’m not comfortable to talk about, and I can imagine if someone out there will have that notion, you know those people who are doing exercises there, those are the people who are HIV-positive and to demoralise the effort that you have and then… you know. This thing I don’t know where the bloody hell does this thing come from.” “No no no, I’m not comfortable exercising in a group. I don’t have a friend; or rather, in my community they know that I fetch medication (referring to ARTs) but I never discuss this matter with anyone.”
Physical health issues	“It’s only one reason, sir. I enjoy it in such a way that, whether it hurts or not, I still do it again. The only thing that distances me from exercise is the pain in my back. There is a new pain that I never ever had before.” “You see, my child, what would interrupt me usually would be the problem I experience in my legs. As we are older, we experience joint pains. Joint pains, muscle tension, when I can’t walk. That would usually prevent me.” “Yes, I definitely enjoyed it (exercise) but the issue is that I got tired. But eventually I realised that I need to get up and start moving. My body is failing me and it’s too heavy to handle. You know if I exercise I feel that my blood is flowing and I feel good and become better.”
Personal circumstances	“The issue is that it is not easy because many people live under one roof. My children, the father of the house. Well the reason could be that I’m tired because the work that I do is quite a lot, you see. Sometimes, when it’s time to go to bed at night, I just want to go to bed, I don’t want to exercise because I’m tired.” “Taking care of my home and my grandchildren means I am busy all day…morning till night. I have no time for exercise.” “There is no urgency for me to exercise because I’m not experiencing complications and I’m feeling fine. I’m not really the running type and it’s not something that I did whilst growing up. Even when I walk, I cannot walk at a fast pace.”
Lack of proper instruction	“That’s my problem. Up until today I love to exercise but I’ve never been instructed thoroughly about it…up to date about the situation. Yes, that is what is worrying me.” “I try to exercise but I don’t feel I am doing the right things, or enough. I try to read up on what to do but it’s not the same. Someone must show me how to do the exercise—the right guidance is important.”
Environmental factors	“If the time permits then I wouldn’t have a problem to attend exercise sessions. Another thing, it would be nice if there was a place where you install equipment in the community so we can exercise together and not me alone because there are many people who are in a similar situation as me—it’s very important.” “I also want to run in a safe area. I don’t run in dangerous places where its dark.”

Theme 3: Proposed structure of physical activity and exercise	
Aerobic exercise	“I realise that walking is quite important, especially if I do go to the centre in Pinetown, I don’t take the lifts. I use the stairs and I find that’s important because last year I had to undergo a major operation, so the doctor wants to know, do you ever go up a flight of stairs? So it’s really important.” “Definitely, the walking has helped my heart to keep on pumping the right way. I’ve seen the difference. I’ve seen it.” “I’m 58 years old and walking has definitely improved mobility and joint pains.”
Aerobic and flexibility	“Well, sometimes, I wake up at 5:00 am in the morning. Then I start around 5:30, am going to 6:00 am. So that by the time my grandchildren are awake I’m already done with my running and stretching.” “Before I go to work, my children and I, we exercise a bit. Maybe we do 25 kicks and we move our bodies counting 25 times for each workout.”
Aerobic and strength	“I still do them, press-ups and walking. I do it every day, not as many press-ups as I used to, but I still do it.” “Running helps me build stamina. When you engage in an exercise like running and some strength activity and you begin to like it you don’t tire easily. Nothing prevents me from running and doing strength exercises.”
Group exercise	“Walking together in a group or exercising together in a group is more beneficial. You forget about exercising and before you know you are done exercising.” “Definitely, I prefer to exercise in a group. That’s where we get encouragement from and forget about the pain and stresses and we all go on this journey of exercising together.” “If you create a programme that I can attend in a group and exercise in this hospital, then I would definitely attend. It will help me to be with my friends too.”

The first theme, motivation for physical activity and exercise, included the sub-themes of: recommendation by a health professional; personal gratification; activities of daily living as a driver; community participation in a religious context; and family responsibility and influence. Both men and women believed that the recommendation of physical activity and exercise by their doctor played an important role in influencing their participation in physical activity and exercise. Personal gratification and the influence of physical activity and exercise in assisting some of the men and women to perform the activities of daily living was a strong motivator, while community participation in a religious context, as well as family responsibility and influence, ensured physical activity and exercise uptake in, and around, the home and in the community.

The second theme, barriers to physical activity and exercise, included the sub-themes of stigma; physical health issues; personal circumstances; lack of proper instruction; and environmental factors. The men and women in our paper believed that HIV-related stigma experienced in their community, and personal physical health issues such as joint pain, were of major concern and hindered their uptake of physical activity. Some participants felt personal circumstances like day-to-day busyness i.e. household chores, personal care and creative activities such as arts and crafts, took up too much of their time and discouraged participation in physical activity and exercise. Other men and women believed it would be beneficial, but lacked an understanding of physical activity (when, where and how much is beneficial), as well as available resources to support such participation.

A third theme emerged around the proposed structure of physical activity and exercise, which the men and women in our paper believed had the most influence on their uptake. It included the sub-themes of aerobic exercise; aerobic exercise and flexibility; aerobic exercise and strength; and group exercise. Most participants believed that walking, and a combination of flexibility and strength activity, was vital to improving their fitness levels. The majority of the participants also saw the benefit of, and advocated for, group exercise, rather than individual exercise.

## Discussion

This study aimed to explore the perceptions of older people living with HIV in a semi-rural area regarding physical activity and exercise. Understanding the perceptions of this specific population about physical activity and exercise can be useful in the development of interventions that are context-specific and feasible for OPLWH.

The theme around the motivation for physical activity and exercise highlighted facilitators to exercise and physical activity participation. The OPLWH in our study believed that a recommendation by their health professional was a catalyst to them being physically active. Some men and women reported that, during consultation with their primary healthcare provider, they were advised to participate in some form of generalised physical activity or exercise. The benefits that were communicated by the healthcare provider included delayed progression of the disease and improved quality of life. This compelled them to engage in some form of physical activity or exercise. However, there was no clear guidance offered by the healthcare team with regards to mode, duration, frequency, and intensity of exercise prescription. Current physical activity recommendations, as described by the American College of Sports Medicine (ACSM) Exercise Management for Persons with Chronic Disease and Disabilities, for people living with HIV, suggest a moderate-intensity aerobic and resistance training regimen [25]. This includes accumulating a total of 150 min of moderate-intensity physical activity a week, as well as two days of full body resistance training, following medical clearance from a primary healthcare provider. The prescription is for people living with HIV, and is not specifically tailored for OPLWH. This could possibly contribute to the hesitancy by healthcare teams to provide clear physical activity and exercise guidelines to the population of OPLWH.

Personal gratification from participation in physical activity was commonly reported by both men and women in our study. Some OPLWH viewed physical activity as a leisure activity rather than a necessity for healthcare. They reported that walking ang fun activities with their much younger grandchildren brought them great joy whilst also helping to reduce stress levels. Both health and well-being are improved in a meaningful, enjoyable, leisure activity, such as physical activity and exercise. It also affords a context for self-validation and expression, self-determination and self-esteem, and creativity; all of which contribute to an improved quality of life and sense of well-being [26–28]. Participation in physical activity and exercise acts as a buffer against the adverse effects of stress on mental and physical health, and serves as a restorative mechanism that helps people transcend negative life events [29, 30]. Studies examining physical activity and exercise levels among older adults are scarce, and research seems to have generally overlooked people growing older with HIV.

Our paper found that the activities of daily living influenced participation in physical activity. Our study was located in a semi-rural area in South Africa where the essential activities of daily living ranged from cooking and cleaning duties in the home, to farming and taking care of livestock. The participants in our study considered these activities as a form of exercise. Yaya et al. [31] assessed the relationship between self-reported difficulty in performing the activities of daily living (ADL’s), health, and quality of life among community-dwelling populations aged 50 years and over in South Africa and Uganda [31]. The study found that only two-fifths of the participants reported having good health, and one-fifth reported enjoying a good quality of life. The analysis revealed disparities of varying degrees in the prevalence of good health and quality of life between participants in both countries, but concluded that having difficulty in the ADL was found to be a significant predictor of poor health and quality of life. Our participants were of the belief that ADLs influenced their participation in physical activity and exercise because it improved their health and overall quality of life.

Religious involvement and family responsibility influenced physical activity in both the men and women in our paper. Other studies in this context highlighted the scarcity of resources, including, transportation and lack of employment [32–34]. This has an influence on the community, as walking becomes the travel mode of necessity. The benefits are sought-after, but the circumstances for choices such as, walking to work, need to be addressed. Older people in a South African context, especially in semi-rural and rural communities, are often responsible for caring for grandchildren while parents work in urban areas [35, 36]. Caring for children requires high levels of physical activity, such as walking to school, playing and helping with grooming and selfcare [37–39]. This circumstance often prevails in poorly resourced communities that lack employment opportunities. Parents travel extensively away from home to find employment, leaving their children in the care of grandparents and older community members for extended periods. This is not often ideal, but within the context of this study, it inadvertently does lead to physical activity and the benefits thereof for OPLWH.

Barriers to participation in physical activity and exercise included stigma. Stigma has been recognized as an important barrier to physical activity and exercise in vulnerable groups [13, 20, 40, 41]. However, in the specific context of OPLWH, there is a lack of research to assess the relationship between physical activity and the perception of HIV-related stigma [42]. HIV-related stigma is defined as social discrediting and devaluation associated with HIV against people perceived to have HIV [43]. Anticipated stigma, enacted stigma and internalized stigma are all mechanisms through which stigma may be experienced by people living with HIV (PLHIV) [44, 45]. Anticipated stigma refers to the expectation of discrimination and prejudice from others, due to one’s HIV status; while enacted stigma refers to experiences of discrimination and prejudice from others due to one’s HIV status [45]. Internalized stigma is related to endorsing negative feelings and beliefs associated with HIV and applying them to oneself [44]. According to Han et al. [46], of the three mechanisms, internalized stigma seems to have the most influence on behaviors and attitudes to physical activity [46]. This can manifest as a reluctance to engage in specific, public forms of activity (e.g. at gyms, in social sports teams), or as avoidance of any physical activity, such as going for a brisk walk. Our participants cited internalized stigma as a major barrier to engaging in some form of physical activity or exercise, especially in a group setting. Akatukwasa et al. [47] also advocate for the further exploration of the connection between levels of internalized stigma and physical activity behavior in PLHIV, in order to better inform physical activity interventions for this population [47]. Developing and implementing physical activity programmes for OPLWH should include factors to mitigate against such stigma in the community. This could include mixed groups of participants within exercise groups, as well as increasing awareness and fostering understanding within such communities. The authors also recognize the need to measure HIV-related stigma in this population, using validated scales similar to those used in younger HIV populations, prior to developing interventions [21, 48, 49].

Our research found that physical health issues and personal circumstances contributed to poor uptake of physical activity and exercise. The link between a decline in physical health and HIV, as well as personal circumstances as a result of HIV, is well documented [50]. The decline in physical health as a result of HIV may be associated with more musculoskeletal problems and subsequent bodily discomfort during participation in physical activity or exercise [51]. This is evident from the experiences of OPLWH in our study. However, it is important to bear in mind that physical activity and exercise enhances physical well-being, which in turn may benefit one’s physical self-perception, especially in HIV populations. Olsen et al. [52] found that higher HIV viral loads were associated with less physical activity and participation in exercise [52]. The authors agreed that a higher HIV load should not be a contra-indication for physical activity, and recommended that patients with higher HI viral load levels perform moderate, instead of high intensity, physical activity. Despite the medical advances in HIV management with antiretroviral therapy, OPLWH appear to experience age-associated comorbidities at a higher rate than their younger counterparts [53]. The physical barriers attributed by our participants to HIV and medication—fatigue, and chronic musculoskeletal pain – are consistently identified as barriers to exercise [20, 54]. To engage OPLWH, exercise interventions must acknowledge and address this issue. The effective management of HIV and support to those infected, together with understanding their personal circumstances, is vital in increasing the opportunity to undertake physical activity.

Other barriers to the uptake of physical activity by the OPLWH in our study included the lack of proper instruction and environmental factors. Many of our OPLWH indicated that they would love to engage in some form of physical activity and exercise, but did not know where, when, or how to begin. One of the men said *“That’s my problem. Up until today I love to exercise but I’ve never been instructed thoroughly about it. Yes, that is what is worrying me.”* Li et al. [55] identified instruction and supervision by a suitably qualified health professional as an important factor to consider when developing and implementing community-based exercise programmes for PLHIV [55]. This greatly enhanced adherence to physical activity and exercise in PLHIV. The men and women in our study believed that environmental factors such as lack of time, and the lack of a conducive environment and facilities, inhibit them from participating in physical activity and exercise. Similar environmental factors were reported by Wright and colleagues [56] when investigating physical activity, exercise, and diet among older Ugandans living with, and without, chronic HIV infection [56]. Most studies conducted in sub-Saharan Africa, although few, have often cited the lack of facilities in rural and semi-rural areas as a barrier to physical activity and exercise adherence.

The theme regarding the proposed structure of physical activity and exercise focused on the belief of OPLWH that the provision of combined activity in the form of aerobic, resistance, flexibility, and balance exercises could be beneficial and should be encouraged. Most of the men and women also preferred group-based exercise rather than individual exercise regimens. Most highlighted the fact that the benefits from social interaction and peer support during group-based exercise was important to them, as outlined by the statement by one of our most senior participants *“Definitely, I prefer to exercise in a group. That’s where we get encouragement and forget about the pain and stresses and we all go on this journey of exercising together.”* The frequency, duration, intensity and time of activity requires further exploration in this population. There seemed to lack of understanding around these parameters and/or how they could be used in prescribed physical activity and exercise to improve their health outcomes. Grace et al. [57] acknowledged that, whilst the primary goals of prescribing exercise in HIV-infected patients are to improve quality of life (QOL), physical tolerance, and neuromuscular function, and to promote long-term exercise compliance, most exercise prescriptions fail to indicate the optimum frequency, duration, intensity and time [57]. This advocates for a closer inspection of the exercise dose–response relationship, which is important when prescribing physical activity and exercise for OPLWH.

## Conclusion

The qualitative nature of this study provides an in-depth understanding of the perceptions of OPLWH about physical activity and exercise. In light of the fact that ART’s have increased the longevity of people living with HIV, such evidence could aid in the development and implementation of an intervention programme to improve the quality of life in OLPWH. Overall, our paper highlighted the interplay between motivating factors and barriers, and the structural components of physical activity and exercise, that can possibly influence the design and implementation of a physical activity intervention programme for OPLWH. Our study also highlights the lack of clear guidance with regards to optimal mode, duration, frequency, and intensity of exercise prescription for this specific population. It also reiterates the authors belief that proper instruction and supervision has the potential improve adherence levels. Recommendations from our study may be used by healthcare professionals and community-based establishments to enhance the design, accessibility and participation in structured physical activity interventions for OPLWH.

## Abbreviations

ART: Antiretroviral therapy
OPLWH: Older people living with HIV
ADL: Activities of daily living
CHC: Community health centre
PLHIV: People living with HIV
QOL: Quality of life

## Declarations

### Availability of data and materials

The materials used and analyzed in this study are available from the corresponding author upon reasonable request.
